# Measles incidence and reporting trends in Germany, 2007–2011

**DOI:** 10.2471/BLT.13.135145

**Published:** 2014-08-15

**Authors:** Anja Takla, Ole Wichmann, Thorsten Rieck, Dorothea Matysiak-Klose

**Affiliations:** a Immunization Unit, Robert Koch Institute, Seestrasse 10, 13353 Berlin, Germany.; b Charité – University Medicine Berlin, Berlin, Germany.

## Abstract

**Objective:**

We aimed to quantify progress towards measles elimination in Germany from 2007 to 2011 and to estimate any potential underreporting over this period.

**Methods:**

We determined the annual incidence of notified cases of measles – for each year – in northern, western, eastern and southern Germany and across the whole country. We then used measles-related health insurance claims to estimate the corresponding incidence.

**Findings:**

In each year between 2007 and 2011, there were 6.9–19.6 (mean: 10.8) notified cases of measles per million population. Incidence decreased with age and showed geographical variation, with highest mean incidence – 20.3 cases per million – in southern Germany. Over the study period, incidence decreased by 10% (incidence rate ratio, IRR: 0.90; 95% confidence interval, CI: 0.85–0.95) per year in western Germany but increased by 77% (IRR: 1.77; 95% CI: 1.62–1.93) per year in eastern Germany. Although the estimated incidence of measles based on insurance claims showed similar trends, these estimates were 2.0- to 4.8-fold higher than the incidence of notified cases. Comparisons between the data sets indicated that the underreporting increased with age and was generally less in years when measles incidence was high than in low-incidence years.

**Conclusion:**

Germany is still far from achieving measles elimination. There is substantial regional variation in measles epidemiology and, therefore, a need for region-specific interventions. Our analysis indicates underreporting in the routine surveillance system between 2007 and 2011, especially among adults.

## Résumé

**Objectif:**

Évaluer les progrès réalisés dans l'élimination de la rougeole en Allemagne entre 2007 et 2011, et estimer toute sous-déclaration potentielle au cours de cette période.

**Méthodes:**

Nous avons déterminé l'incidence annuelle des cas déclarés de rougeole (pour chaque année) dans le nord, l'ouest, l'est et le sud de l'Allemagne et dans tout le pays. Nous avons ensuite utilisé les demandes de remboursement à l'assurance maladie liées à la rougeole pour estimer l'incidence correspondante.

**Résultats:**

Chaque année entre 2007 et 2011, il y a eu entre 6,9 et 19,6 (moyenne: 10,8) cas déclarés de rougeole par million d'habitants. L'incidence diminuait avec l'âge et montrait une variation géographique, avec l'incidence moyenne la plus élevée (20,3 cas par million d'habitants) dans le sud de l'Allemagne. Pendant la période de l'étude, l'incidence a diminué de 10% (rapport de taux d'incidence, RTI: 0,90; intervalle de confiance à 95%, IC 95%: 0,85–0,95) par an dans l'ouest de l'Allemagne mais a augmenté de 77% (RTI: 1,77; IC 95%: 1,62–1,93) par an dans l'est de l'Allemagne. Bien que l'incidence estimée de la rougeole basée sur les demandes de remboursement à l'assurance maladie ait montré des tendances similaires, ces estimations étaient 2,0 à 4,8 fois supérieures à l'incidence des cas déclarés. Les comparaisons entre les ensembles de données ont indiqué que la sous-déclaration augmentait avec l'âge et était généralement plus faible les années où l'incidence de la rougeole était élevée, que lors des années à incidence faible.

**Conclusion:**

L'Allemagne est toujours loin d'avoir éliminé la rougeole. Il existe des variations régionales substantielles en matière d'épidémiologie de la rougeole et, par conséquent, un besoin d'interventions spécifiques aux régions. Notre analyse indique une sous-déclaration dans le système de surveillance de routine entre 2007 et 2011, en particulier chez les adultes.

## Resumen

**Objetivo:**

Pretendimos cuantificar el progreso hacia la eliminación del sarampión en Alemania de 2007 a 2011 y estimar cualquier falta de notificación en el periodo.

**Métodos:**

Se determinó la incidencia anual de casos notificados de sarampión para cada año en el norte, el oeste, el este y el sur de Alemania, así como en todo el país. A continuación, se emplearon las reclamaciones de seguros médicos relacionadas con el sarampión para estimar la incidencia correspondiente.

**Resultados:**

Cada año entre 2007 y 2011, hubo entre 6,9 y 19,6 (media: 10,8) casos notificados de sarampión por millón de habitantes. La incidencia disminuyó con la edad y mostró variación geográfica, con mayor incidencia media (20,3 casos por millón) en el sur de Alemania. Durante el periodo del estudio, la incidencia se redujo en un 10 % (razón de tasas de incidencia, IRR: 0,90; Intervalo de confianza del 95 %, IC: 0,85-0,95) al año en el oeste de Alemania, pero aumentó en un 77 % (IRR: 1,77; IC del 95 %: 1,62-1,93) al año en el este de Alemania. Si bien la incidencia estimada del sarampión basada en las reclamaciones de seguros mostró tendencias similares, estas estimaciones eran de 2,0 a 4,8 veces más elevadas que la incidencia de casos notificados. Las comparaciones entre los conjuntos de datos indicaron que la falta de notificación aumentó con la edad y que fue generalmente menor durante los años en que la incidencia del sarampión fue alta que en los años de incidencia baja.

**Conclusión:**

Alemania sigue lejos de lograr la erradicación del sarampión. Existe una variación regional considerable en la epidemiología del sarampión y, por lo tanto, se requieren intervenciones específicas en cada región. Nuestro análisis señala una falta de notificación en el sistema de vigilancia sistemática entre 2007 y 2011, sobre todo entre los adultos.

## ملخص

**الغرض:**

استهدفنا تحديد درجة التقدم نحو استئصال الحصبة في ألمانيا في الفترة من 2007 إلى 2011 ولتقدير أي نقص محتمل في الإبلاغ على مدار الفترة ذاتها.

**الطريقة:**

قمنا بتحديد معدل الإصابة السنوي لحالات الحصبة التي تم الإبلاغ عنها - لكل عام - في شمال وغرب وشرق وجنوب ألمانيا وبين مختلف مناطق البلد بالكامل. واستخدمنا بعد ذلك مطالبات التأمين الصحي ذات الصلة بالحصبة لتقدير معدل الإصابة المقابلة.

**النتائج:**

تراوحت حالات الحصبة التي تم الإبلاغ عنها في كل عام في الفترة من 2007 إلى 2011 من 6.9 إلى 19.6 حالة لكل مليون شخص (المتوسط: 10.8 حالة). وانخفض معدل الإصابة مع التقدم في العمر وأظهر تبايناً جغرافياً وبلغ أعلى متوسط لمعدل الإصابة 20.3 حالة لكل مليون شخص في جنوب ألمانيا. وعلى مدار فترة الدراسة، انخفض معدل الإصابة بنسبة 10 % (نسبة معدل الإصابة: 0.90؛ فاصل الثقة 95 %، فاصل الثقة: من 0.85 إلى 0.95) سنوياً في غرب ألمانيا، غير أنها ازدادت بنسبة 77 % (نسبة معدل الإصابة: 1.77؛ فاصل الثقة 95 %: من 1.62 إلى 1.93) سنوياً في شرق ألمانيا. وعلى الرغم من الاتجاهات المشابهة التي أظهرتها تقديرات معدل الإصابة بالحصبة المستندة إلى مطالبات التأمين، شهدت هذه التقديرات زيادة تراوحت من 2.0 إلى 4.8 ضعف معدل الإصابة للحالات التي تم الإبلاغ عنها. وأشارت المقارنات بين مجموعات البيانات إلى ازدياد نقص الإبلاغ مع التقدم في العمر وانخفاضه عموماً في السنوات التي يكون فيها معدل الإصابة بالحصبة مرتفعاً عن السنوات التي ينخفض فيها معدل الإصابة بالحصبة.

**الاستنتاج:**

ما زالت ألمانيا بعيدة عن تحقيق استئصال الحصبة. وهناك تباين جغرافي كبير في الوضع الوبائي للحصبة ومن ثم هناك حاجة لإجراء تدخلات خاصة بالإقليم. ويشير تحليلنا إلى نقص الإبلاغ في نظام الترصد الروتيني في الفترة من 2007 إلى 2011، لاسيما بين البالغين.

## 摘要

**目的:**

我们旨在量化2007年到2011年德国在麻疹消除方向上取得的进展，并估算在此期间的任何潜在漏报。

**方法:**

我们确定德国北部、西部、东部和南部以及整个国家通告麻疹病例每年发病率。然后，我们使用与麻疹相关的医疗保险索赔情况来估计相应的发病率。

**结果:**

在2007和2011年之间的每一年中，每百万人口中有6.9-19.6（平均：10.8）个通告麻疹病例。发病率随年龄下降，并显示出地理差异，以德国南部平均发病率为最高，达到每百万人口20.3个病例。在研究期间，德国西部发病率每年下降10%（发病率比，IRR：0.90；95%置信区间，CI：0.85-0.95），但在德国东部每年增加77%（IRR：1.77；95% CI：1.62-1.93）。尽管基于保险索赔的麻疹估计发病率显示出相似的趋势，但是这些估计比通告病例发病率高2.0-4.8倍。数据集之间的比较表明，漏报随年龄增加，并且少报的情况在麻疹发病率高的年份通常比发病率低的年份更少。

**结论:**

德国还远未实现消灭麻疹的目标。麻疹流行病学特点存在巨大的地区性差异，因此需要地区针对性的干预措施。我们的分析表明在2007年至2011年之间日常监测系统存在漏报，在成年人当中尤其如此。

## Резюме

**Цель:**

Нашей целью являлось измерить прогресс в деле ликвидации кори в Германии за период 2007—2011 гг. и оценить любое потенциальное занижение данных о заболевании за этот же период.

**Методы:**

Мы определили ежегодные уровни заболеваемости на основе числа зарегистрированных случаев кори для каждого года по регионам — в северной, западной, восточной и южной Германии и по всей стране. Затем мы сравнили эти данные с числом страховых случаев, зарегистрированных в системе медицинского страхования, также с целью оценить соответствующие уровни заболеваемости.

**Результаты:**

В течение каждого года за период 2007—2011 гг. было зарегистрировано 6,9—19,6 (в среднем: 10,8) случаев кори на один миллион человек. Заболеваемость снижалась с возрастом и показала географическую изменчивость, при этом самая высокая средняя заболеваемость — 20,3 случаев на миллион человек — была зарегистрирована в южной Германии. За период исследования заболеваемость снизилась на 10% (показатель роста заболеваемости, IRR: 0,90; 95% доверительный интервал, ДИ: 0,85–0,95) за год в западной Германии, но возросла на 77% (IRR: 1,77; 95% ДИ: 1,62–1,93) за год в восточной Германии. Хотя уровень заболеваемости корью на основе страховых случаев показал аналогичные тенденции, число случаев, по данным страховых компаний, было в 2,0—4,8 раз выше, чем число зарегистрированных случаев в системе эпиднадзора. Сравнение между наборами данных показало, что занижение увеличивалось с возрастом и в целом было меньше в годы, когда заболеваемость корью была высокой, по сравнению с годами с низким уровнем заболеваемости.

**Вывод:**

Германия по-прежнему далека от достижения полной ликвидации кори. Существует немало региональных различий в эпидемиологии кори и, следовательно, необходимо принимать меры в соответствии со спецификой региона. Наш анализ показывает занижение данных о заболеваемости в системе текущего эпиднадзора за период 2007—2011 гг., особенно среди взрослых.

## Introduction

Measles is a viral disease that can lead to severe complications such as encephalitis, pneumonia and death.^1^ All of the countries within the World Health Organization (WHO) European Region have committed to the elimination of measles by 2015. WHO defines successful elimination of measles as “the absence of endemic measles … cases in a defined geographical area for a period of at least 12 months, in the presence of a well-performing surveillance system”.^2^ An incidence of less than one measles case per million population has been suggested as a useful indicator of the progress made towards the disease’s elimination.^2^ In the years 2012 and 2013, only about a third of the countries in the WHO European Region recorded incidence below this threshold.^2^^,^^3^ In early 2014, the WHO Strategic Advisory Group of Experts concluded that measles elimination will not be achieved in the WHO European Region by 2015.^4^

Measles has been a notifiable disease in Germany since 2001. Free-of-charge measles vaccination was introduced into the routine childhood vaccination schedule in 1970 in the German Democratic Republic and in 1974 in the Federal Republic of Germany. In 2010, such vaccination was also made available to incompletely vaccinated adults living in Germany. Despite the access to measles vaccine, measles outbreaks still occur frequently in Germany. In 2006, for example, more than 1500 cases were recorded in an outbreak in the federal state of North Rhine-Westphalia.^5^^,^^6^^,^^7^ Historically, health-related issues in Germany – including the implementation of vaccination recommendations and measures for disease control – have been the responsibility of the country’s 16 federal states.^8^ This is one of the reasons why vaccination coverage in Germany often differs between federal states. Most importantly, measles vaccination was mandatory in the German Democratic Republic,^9^ and – despite abolishment of all mandatory vaccinations after reunification in 1990 – coverage remained higher in eastern Germany than elsewhere in the country.^10^ In comparison, federal states in southern Germany have had particularly low levels of measles vaccination coverage at school entry and showed a delayed increase in two-dose vaccination coverage after the two-dose recommendation was endorsed in 1991. For example, in 2005 and 2011, coverages for the second dose at school entry were 87.1% and 96.1%, respectively, in Mecklenburg-Western Pomerania, eastern Germany, but only 67.9% and 89.8%, respectively, in Bavaria, southern Germany.^11^^,^^12^

Although an effective system of routine surveillance is essential for monitoring and documenting the progress made towards measles elimination,^2^ most countries rely on passive surveillance systems that are prone to underreporting.^3^^,^^13^^,^^14^ As they strive to achieve measles elimination, countries need to evaluate the sensitivity of their measles surveillance and identify the areas that need improvement.^3^ Health-insurance claims recorded by the Associations of Statutory Health Insurance Physicians (ASHIP) provide an alternative data set for estimating outpatient disease incidence and vaccination coverage.^15^ Approximately 85% of Germany’s inhabitants are covered by statutory health insurance. ASHIP data have already been used to estimate disease burden or incidence of herpes zoster^16^ and mumps^17^ and to assess potential underreporting during the measles outbreak that occurred in North Rhine-Westphalia in 2006.^7^

In this study, we aimed to determine the progress that Germany has made towards measles elimination by estimating annual measles incidence, at national and subnational level, for the years 2007–2011. By comparing the measles incidence of notified cases with the corresponding incidence estimated from ASHIP data, we also aimed to evaluate the level of potential underreporting represented by the data from the mandatory notification system.

## Methods

### Definitions

In our analyses of the mandatory notification data, a measles case was defined as a person reported within the national surveillance system with laboratory-confirmed measles – i.e. positive for measles-specific immunoglobulin (Ig) M, showing a substantial increase in measles-specific IgG or positive for measles RNA (ribonucleic acid) in a polymerase chain reaction – and/or with the clinical symptoms of measles – i.e. a maculo-papular rash and fever for at least three days, plus at least one of the following: cough, catarrh, Koplik’s spots and conjunctivitis.

Within the ASHIP data set, a measles case was defined as a person who had been diagnosed by a physician with a measles-related code from the tenth revision of the *International Classification of Diseases and Related Health Problems, 10th Revision* (*ICD-10*) – i.e. B05.0, B05.1, B05.2, B05.3, B05.4, B05.8 or B05.9, indicating cases of measles with encephalitis, meningitis, pneumonia, otitis media, intestinal complications, other complications or no complications, respectively.

For some of our analyses, we divided Germany into four areas: northern – comprising the federal states of Bremen, Hamburg, Lower Saxony and Schleswig-Holstein; western – Hesse, North Rhine-Westphalia, Rhineland-Palatinate and Saarland; eastern – Berlin, Brandenburg, Mecklenburg-Western Pomerania, Saxony, Saxony-Anhalt and Thuringia; and southern – Bavaria and Baden-Württemberg. In 2011, the northern, western, eastern, and southern areas had populations of approximately 13 million, 29 million, 16 million and 23 million, respectively.^18^

### Mandatory notification data

We extracted national data on the measles cases recorded by the mandatory notification system – for the years 2007–2011 – using the SurvNet@RKI software package.^19^ SurvNet@RKI – or SurvNet@RKI-like softwares – are used by local health authorities in Germany to report anonymized information on inpatient and outpatient cases with notifiable diseases to the relevant state health authorities and on to the national health authority. For each reported case, the data set included the case’s month and year of birth, sex and vaccination status, the week and year of the notification and whether the case was hospitalized. To be able to make comparisons with the ASHIP data, we excluded all hospitalized cases from the notification data, all cases reported from the federal state of Hesse and cases reported in 2007 from the federal state of Baden-Württemberg.

### ASHIP data

In Germany, once every quarter, physicians accredited with statutory health insurances send their reimbursement claims for provided ambulatory medical services to their corresponding regional ASHIP. For each case, the ASHIP data set contained the patient’s unique identifier, month and year of birth, sex, ICD-10 code, quarter and year of diagnosis, reliability of diagnosis – suspected, confirmed, recovered or excluded – and type of diagnosis – current state, previous state, unknown or not provided.

We analysed measles-related reimbursement claims made between 1 January 2007 and 31 December 2011. We excluded incomplete data from the federal state of Hesse and data on cases diagnosed in 2007 in the federal state of Baden-Württemberg. The final data set covered 68% and 79% of the total population living in Germany in 2007 and 2008–2011, respectively.

We only included diagnoses coded as confirmed and current in our incidence estimates. We generally used a four-step algorithm to limit the data set to a single diagnosis for each patient’s unique identifier (Table 1). However, for the data from Bavaria, Rhineland-Palatinate and – for the period 2008–2011 – parts of North Rhine-Westphalia, step 3 had to be omitted because the corresponding data did not contain information on type of diagnosis.

**Table 1:** Cleaning of data from Associations of Statutory Health Insurance Physicians, Germany, 2007–2011

Cleaning step	Procedure	No. of records remaining	
		Bavaria, Rhineland-Palatinate, parts of North Rhine-Westphalia	Other federal states^a^
Baseline	–	23 084	46 766
1	Exclusion of incompatible or implausible coding combinations for reliability of diagnosis	22 950	46 615
2	Exclusion of observations with reliability of diagnosis coded as suspected, excluded or recovered	4 696	10 396
3	Exclusion of observations with type of diagnosis coded as previous state, unknown or not provided	NA	6 869
4	Limitation to the most severe ICD-10 diagnostic code assigned to each patient identification number^b^	3 046	5 607

ICD-10: *International Classification of Diseases and Related Health Problems, 10th revision*; NA: not applicable.

^a^ Remaining parts of North Rhine-Westphalia plus the federal states of Baden-Württemberg, Berlin, Brandenburg, Bremen, Hamburg, Lower Saxony, Mecklenburg- Western Pomerania, Saarland, Saxony, Saxony-Anhalt, Schleswig-Holstein and Thuringia.

^b^ The diagnoses were measles with – in descending order of severity – encephalitis, meningitis, pneumonia, intestinal complication, otitis media, other complication and no complication.

### Statistical analysis

Incidence, with 95% confidence intervals (CI), was calculated either as the number of notified cases or outpatient cases per million total population – for the mandatory notification data – or as the number of outpatient cases per million residents with statutory health insurance – for the ASHIP data.^18^^,^^20^ We calculated incidence ratios by dividing the incidence estimated from the ASHIP data by the incidence derived from the notification data. We used Poisson regression to determine the temporal trends in incidence, as incidence rate ratios (IRR). Statistical analysis was performed by using Stata, version 12.1 (StataCorp. LP, College Station, United States of America).

## Results

### Notified measles incidence

The demographics of all measles cases notified in Germany between 2007 and 2011 are given in Table 2. The total numbers of cases and corresponding incidence are shown – stratified by year, age group and geographical area – in Table 3. Vaccination status was available for 4142 (93.3%) of the 4440 notified cases: 3730 (90.1%) were unvaccinated and 322 (7.8%), 84 (2.0%) and 6 (0.1%) had received one dose, two doses and at least three doses of measles-virus-containing vaccine, respectively.

**Table 2:** Demographics of measles cases, Germany, 2007–2011

Demographic	Mandatory notification data		ASHIP data on outpatients (*n* = 8653)
	All cases (*n* = 4440)	Outpatients (*n* = 3364)	
**Males, no. (%)**	2151 (48)	1646 (49)	3828 (44)
**Age (years)**			
Median	14	11	15
25th percentile	6	6	6
75th percentile	20	16	37
**Complications, no. (%)**			
Pneumonia	85 (1.9)	21 (0.6)	173 (2.0)
Otitis media	71 (1.6)	44 (1.3)	678 (7.8)
Encephalitis	4 (0.1)	NA^a^	NA^a^

ASHIP: Associations of Statutory Health Insurance Physicians; NA: not applicable.

^a^ Not calculated because the majority of cases require hospitalization.

**Table 3:** Measles incidence according to mandatory notification data, Germany, 2007–2011

Category	No. of cases	Annual incidence^a^ (95% CI)
**Year**		
2007	566	6.9 (6.3–7.5)
2008	915	11.2 (10.4–11.9)
2009	571	7.0 (6.4–7.6)
2010	780	9.5 (8.9–10.2)
2011	1608	19.6 (18.7–20.6)
2007–2011	4440	10.8 (10.5–11.2)
**Age (years)**		
< 1	207	61.4 (53.3–70.3)
0–9	1772	50.1 (47.8–52.5)
10–19	1557	37.5 (35.7–39.4)
20–29	597	12.1 (11.1–13.1)
30–39	338	6.7 (6.0–7.4)
40–49	140	2.0 (1.7–2.4)
≥ 50	36	0.2 (1.5–3.0)
**Area^b^**		
Northern	574	8.7 (8.0–9.4)
Western	1047	7.2 (6.8–7.7)
Eastern	455	5.5 (5.1–6.1)
Southern	2361	20.3 (19.5–21.1)

CI: confidence interval.

^a^ Cases per million population.

^b^ Area information was missing for three cases.

### Comparison of data

**Fig. 1:**
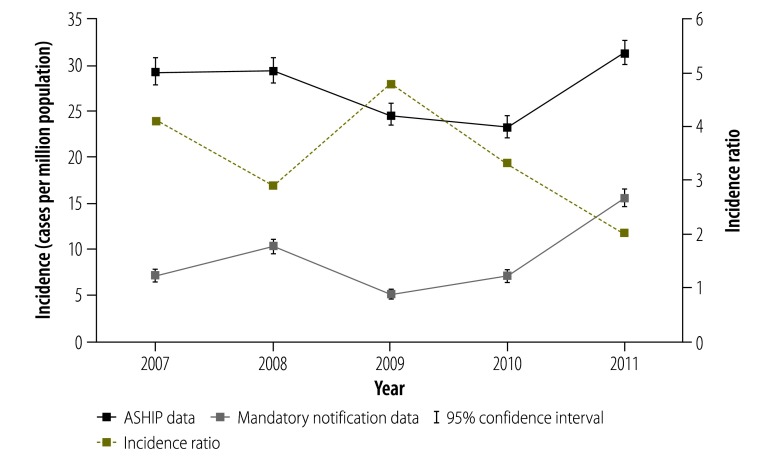
Annual incidence of outpatient measles cases, Germany, 2007–2011

Note: Incidence was estimated using two data sources – the mandatory notification system and the Associations of Statutory Health Insurance Physicians (ASHIP) – and expressed as the number of cases per million population and the number of cases per million residents with statutory health insurance, respectively.

**Fig. 2:**
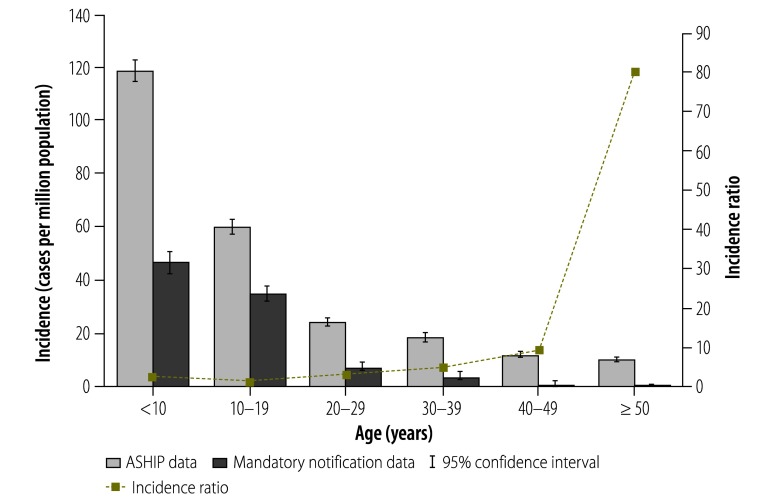
Age-specific annual incidence of outpatient measles cases, Germany, 2007–2011

Note: Incidence was estimated using two data sources – the mandatory notification system and the Associations of Statutory Health Insurance Physicians (ASHIP) – and expressed as the number of cases per million population and the number of cases per million residents with statutory health insurance, respectively.

Results of the ASHIP data cleaning are displayed in Table 1. The demographics of outpatient measles cases and ASHIP cases between 2007 and 2011 are given in Table 2. For Germany as a whole, our estimate of the mean annual incidence – based on ASHIP data – was more than threefold higher than the annual incidence of notified measles cases: 27.5 (95% CI: 27.0–28.1) versus 9.1 (95% CI: 8.8–9.4) cases per million population. Fig. 1 depicts the annual incidence estimated using each data set and the corresponding incidence ratios. Over this five-year time period, incidence estimated from the ASHIP data was 2.0- to 4.8-fold higher than incidence based on the notification data. Fig. 2 shows mean annual incidence and incidence ratios for the two outpatient data sets stratified by age group. Although measles incidence decreased with age, the incidence ratios increased with age.

Incidence ratios were calculated by dividing the incidence estimated from the ASHIP data by the incidence derived from the notification data.

Incidence ratios were calculated by dividing the incidence estimated from the ASHIP data by the incidence derived from the notification data.

**Fig. 3:**
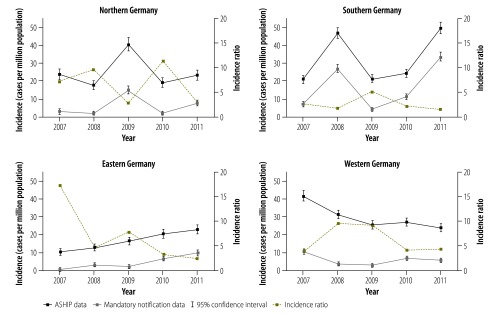
Annual incidence of outpatient measles cases in four areas of Germany, 2007–2011

Note: Incidence was estimated using two data sources – the mandatory notification system and the Associations of Statutory Health Insurance Physicians (ASHIP) – and expressed as the number of cases per million population and the number of cases per million residents with statutory health insurance, respectively.

The measles incidence based on either data set differed substantially between geographical areas (Fig. 3). In general, the incidence ratios were smaller in the years with high incidence of measles than in low-incidence years (Fig. 3). For the western area, we observed a significant decreasing incidence trend between 2007 and 2011, both in the estimates based on mandatory notification data (–10% per year; IRR: 0.90; 95% CI: 0.85–0.95) and in those based on the ASHIP data (–13% per year; IRR: 0.87; 95% CI: 0.85–0.90). In contrast, the eastern area showed a significant increasing trend in the estimates based on the mandatory notification data (+77% per year; IRR: 1.77; 95% CI: 1.62–1.93) as well as in those based on the ASHIP data (+22% per year; IRR: 1.22; 95% CI: 1.17–1.27). Due to the nonlinear patterns observed in measles incidence in the northern and southern areas – which were caused by several large outbreaks – it was not meaningful to perform trend analysis for these two areas.

Incidence ratios were calculated by dividing the incidence estimated from the ASHIP data by the incidence derived from the notification data.

The northern area comprises the federal states of Bremen, Hamburg, Lower Saxony and Schleswig-Holstein. The western area comprises the federal states of North Rhine-Westphalia, Rhineland-Palatinate and Saarland. The eastern area comprises the federal states of Berlin, Brandenburg, Mecklenburg-Western Pomerania, Saxony, Saxony-Anhalt and Thuringia. The southern area comprises the federal states of Baden-Württemberg and Bavaria.

## Discussion

The annual incidence of notified measles cases in Germany exceeded the WHO progress indicator – of less than one notified case per million – throughout our five-year study period. The highest incidence of notified cases was observed in southern Germany and, generally, among infants, older children and adolescents. It appears that, despite the gradual increase in nationwide coverage with two-dose measles vaccination in Germany – from 21.1% at school entry in 1998–2001^21^ to 92.1% in 2011^12^ – population immunity has never been sufficient to stop the circulation of measles virus in the country as a whole or in each area of the country. Moreover, analysis of the ASHIP data on reimbursements for measles diagnoses indicates that there are approximately three cases of measles in Germany for every case of the disease that is formally notified.

In our analysis we observed distinct regional differences in measles incidence and trends. These differences can be largely explained by local differences in vaccination coverage and population immunity.^8^^,^^9^ Several possible reasons for these differences in vaccine uptake have already been discussed.^22^^–^^25^ For example, there is a fairly recent – i.e. pre-1990 – history of mandatory measles vaccination in eastern Germany. Compared with the other areas, individuals who live in southern Germany are more likely to be anthroposophic or vaccine-sceptic or both. The existence of vaccine-sceptic physicians in some districts and general shortages of physicians in certain rural districts may also cause geographical gaps in vaccine coverage. In a review in 1988 of more than 86 000 immunization records of Bavarian children aged 10–12 years, only 54.8% of the children were found to have received one dose of measles vaccine.^26^ Due to a lack of concerted catch-up campaigns of measles vaccination, it seems likely that the many children who were unvaccinated when aged 10–12 years in 1988 still are unvaccinated. Low vaccine coverage in southern Germany presumably contributed to several major outbreaks of measles in the area over the previous years. Analysis of notification data indicates that Bavaria was hit by a major measles outbreak every two–three years between 2001 and 2013.^27^^,^^28^ The absence of characteristically prolonged interepidemic spacing intervals suggests that Bavaria has probably not yet reached a pre-elimination phase.^29^ In contrast, we found that measles incidence in the western area followed a constantly decreasing trend between 2007 and 2011. This encouraging trend may be associated with concerted programmes of health education and a catch-up campaign of measles vaccination, as well as the efforts to increase vaccination coverage at school entry that followed the large-scale outbreak of measles that occurred in North Rhine-Westphalia in 2006.^8^ The proportion of children in North Rhine-Westphalia who had received a second dose of measles vaccine at school entry increased from 74.7% in 2005 to 94.1% in 2011.^11^^,^^12^

Despite having the highest rates of vaccination coverage in Germany, the eastern area experienced ever increasing incidence of measles between 2007 and 2011. Possible explanations could be the influx of new residents from German areas with traditionally lower coverage rates – e.g. into the former East Berlin and the districts surrounding Berlin – and a temporal decrease in the area’s vaccination coverage rates in the five–six years after reunification.^9^^,^^30^

Our comparison of estimated incidence based on ASHIP data with the – markedly lower – values based on Germany’s system of mandatory disease notification indicates a substantial level of underreporting in the established routine surveillance system. The ASHIP data showed similar temporal and regional trends in measles incidence to those detected in the notification data. As previously reported,^7^ the apparent level of underreporting tended to be lower in high-incidence or outbreak years than in other years. Perhaps physicians consider notification of sporadic measles cases as less important than the notification of outbreak-associated cases. An alternative explanation may be that, during measles outbreaks, physicians are reminded of their notification duties through intensified media coverage and increases in the measles-related information provided by public health authorities and physicians’ associations.

The magnitude of underreporting in our analysis increased with the age of the case – as also observed in the potential underreporting of mumps cases in Germany.^17^ Perhaps paediatricians are more aware of the need to notify – or are more accustomed to notifying – local health authorities of cases of measles – and other infectious diseases that predominantly occur or used to occur in childhood – than physicians who treat adult patients.

Although analysis of both the notification data and the ASHIP data indicated that measles incidence in Germany decreased with age, our estimates based on ASHIP data of measles incidence among adults aged at least 40 years were still surprisingly high. The German Standing Committee on Vaccination does not recommend measles vaccination for any individuals born before 1970 – i.e. the year when measles vaccination was introduced into any part of Germany – because such individuals are assumed to have acquired natural immunity to measles when nobody in Germany was vaccinated.^30^^,^^31^ The American Advisory Committee on Immunization Practices set the corresponding cut-off at 1957 – i.e. six years before measles vaccine was introduced in the United States of America.^32^ In a measles outbreak that occurred in Germany in 2013 and involved more than 1600 notified cases, approximately 9% of the cases were aged at least 40 years.^28^ In addition to our findings, the analysis of recent epidemiological data and population-based surveys of serological immunity against measles will inform future discussions within the German Standing Committee on Vaccination when any re-evaluation of the current vaccination strategy is considered.

Our study had several limitations. The assessment of measles incidence from cases of the disease reported to a routine notification or surveillance system is prone to underreporting.^3^^,^^13^^,^^14^ However, as a passive surveillance system, the German system for disease notification is not designed to detect every single case – and therefore has limitations when being used for the verification and documentation of measles elimination. Due to its structure, the use of ASHIP data for estimating potential underreporting of measles in Germany also has some limitations. First of all, ASHIP data are not collected for surveillance purposes but to ensure that physicians are reimbursed for provided medical services. We only included cases coded as confirmed in our analysis of the ASHIP data and therefore the resultant estimates of measles incidence are probably conservative – although a case coded as confirmed does not require laboratory confirmation. Moreover, there are no standardized guidelines for a physician faced with coding a case as confirmed or suspected. ASHIP data have only recently become available for studies of vaccine-preventable diseases in Germany^7^^,^^16^^,^^17^ and their use in such studies has still to be systematically validated. In our analysis at regional level, the variations and trends in measles incidence seen in the notification data were similar to those seen in the ASHIP data. The ASHIP data therefore appear to be useful at least in monitoring trends. As the ASHIP data only cover ambulatory cases of measles, incidence of measles may be higher than those indicated by the data. In Germany, however, 76% of measles cases notified between 2007 and 2011 were treated as outpatients and other cases might have been seen as ambulatory cases before being hospitalized. When we assessed underreporting in the routine surveillance system, we had to restrict our analysis to outpatient cases; the degree of underreporting of hospitalized measles cases therefore remains unknown.

Without concerted efforts to close immunity gaps, Germany will not achieve the goal of measles elimination by 2015. Regional data on measles-related knowledge and attitudes to measles and measles vaccination in the general population and among physicians – as well as on other potential barriers – would be helpful for understanding the regional differences seen in measles incidence and for tailoring elimination measures to the target area. ASHIP records appear to be a valuable source of complementary data, especially for estimating the level of underreporting in the routine notification system. Enhanced efforts to remind physicians of all specialties about their duty to report suspected and confirmed measles cases – both during outbreaks and, especially, during non-outbreak periods – will be crucial in documenting Germany’s future progress towards measles elimination.
